# Widely Targeted Metabolomics Analysis to Reveal Transformation Mechanism of *Cistanche Deserticola* Active Compounds During Steaming and Drying Processes

**DOI:** 10.3389/fnut.2021.742511

**Published:** 2021-10-14

**Authors:** Ziping Ai, Yue Zhang, Xingyi Li, Wenling Sun, Yanhong Liu

**Affiliations:** College of Engineering, China Agricultural University, Beijing, China

**Keywords:** *cistanche deserticola*, widely targeted metabolomics, steaming, drying, active compounds, formation mechanism

## Abstract

*Cistanche deserticola* is one of the most precious plants, traditionally as Chinese medicine, and has recently been used in pharmaceutical and healthy food industries. Steaming and drying are two important steps in the processing of *Cistanche deserticola*. Unfortunately, a comprehensive understanding of the chemical composition changes of *Cistanche deserticola* during thermal processing is limited. In this study, ultra-performance liquid chromatography-tandem mass spectrometry (UHPLC-MS/MS)-based widely targeted metabolomics analysis was used to investigate the transformation mechanism of *Cistanche deserticola* active compounds during steaming and drying processes. A total of 776 metabolites were identified in *Cistanche deserticola* during thermal processing, among which, 77 metabolites were differentially regulated (*p* < *0.05*) wherein 39 were upregulated (UR) and 38 were downregulated (DR). Forty-seven (17 UR, 30 DR) and 30 (22 UR, 8 DR) differential metabolites were identified during steaming and drying, respectively. The most variation of the chemicals was observed during the process of steaming. Metabolic pathway analysis indicated that phenylpropanoid, flavonoid biosynthesis, and alanine metabolism were observed during steaming, while glycine, serine, and threonine metabolism, thiamine metabolism, and unsaturated fatty acid biosynthesis were observed during drying. The possible mechanisms of the chemical alterations during thermal processing were also provided by the Kyoto Encyclopedia of Genes and Genomes (KEGG) pathway analysis. Furthermore, the blackening of the appearance of *Cistanche deserticola* mainly occurred in the steaming stage rather than the drying stage, which is associated with the metabolism of the amino acids. All results indicated that the formation of active compounds during the processing of *Cistanche deserticola* mainly occurred in the steaming stage.

## Introduction

*Cistanche deserticola*, belonging to the orobanchaceae family, is one of the most famous tonic medicines and is mainly distributed in the tropical and subtropical regions of the world, such as China, Iran, India, Mongolia, and so on (1–3). *Cistanche deserticola* serves as one of the most commonly utilized herbal medicines for the treatments of kidney deficiency, impotence, female infertility, morbid leucorrhea, profuse metrorrhagia, and senile (4, 5). Modern pharmacological research showed that *Cistanche deserticola* has the effects of improving immunity, anti-fatigue, anti-aging, and enhancing learning and memorization ability (6). Owing to these health benefits, *Cistanche deserticola* tea made from its stem tubers has been developed as a nourishing supplement and is being increasingly favored by consumers. The active ingredients of *Cistanche deserticola* have been shown to be responsible for its medicinal functions (7). Some active ingredients in *Cistanche deserticola*, such as phenylpropanoids (for example phenylethanoid glycosides), flavonoids, polysaccharides, oligosaccharides, iridoids, and lignans have been reported in the previous studies (6, 8).

Due to the perishable and seasonal features, the all-year-round supply of fresh *Cistanche deserticola* is unavailable, accordingly, the processed *Cistanche deserticola* becomes the main consumption form. The quality of *Cistanche deserticola* is dependent on many factors, such as climate, habitats, hosts, harvest time, processing technology, and position on the plant, among which processing technology is particularly important (4). Steaming and drying are two important steps in the processing of *Cistanche deserticola*. Usually, the harvested *Cistanche deserticola* rhizome was steamed in a steaming boiler at 93°C for 30 min and then dried at 60°C until the moisture content of 10% on a wet basis (w.b.) (9). Previous studies have shown that steaming can promote the accumulation of active ingredients in *Cistanche deserticola*, such as phenylethanoid glycosides, soluble sugars, and polysaccharides, accompanied by blackening of appearance color (9–11). However, most of the previous studies focused on certain specific compounds, very rare research are about the changes in all the chemical compounds and the metabolite conversion mechanism during processing. Therefore, it is necessary to clarify the metabolite changes of *Cistanche deserticola* in different processing stages.

Metabolomics is usually applied to qualitative and quantitative analysis of all small molecules (namely, targeted and non-targeted compounds) detected in the sample (12). Analysis of changes in various chemical components during food processing helps to deepen the understanding of the mechanism of chemical component transformation in food processing (12). In recent years, metabolomics has also been applied to the study of *Cistanche deserticola* for the discrimination of different parts (11) and different *Cistanche deserticola* species (13). The detection methods of metabolites in these studies were mostly based on targeted and non-targeted metabolomics. Among them, targeted metabolomics is based on standard products, with high data accuracy and reliability, however, limited coverage of metabolites. Targeted metabolomics is an important part of metabolomics research, it is the targeted and specific detection and analysis for specific metabolite groups, rather than all the components in the sample. Non-targeted metabolomics technology can qualitatively determine the metabolites based on existing databases, with high coverage of compounds, however, low accuracy. The key metabolites must be confirmed by standard products (14). Widely targeted metabolomics is a new technology that integrates the advantages of non-target and targeted metabolites detection technologies to achieve wide coverage, high throughput, and sensitivity (15). Consequently, this technology has been widely used in the study of ingredient changes in different materials during processing, such as active ingredients in functional foods by different processing methods (16), flavonoids and phenylpropanoids compounds in Chinese water chestnut processed with different methods (17), rice yellowing mechanism during yellowing process (18), and the formation mechanism of characteristic non-volatile chemical constitutes during oolong tea manufacturing process (19). Therefore, it is theoretically feasible to use widely targeted metabolite technology to study the mechanism of the conversion of active ingredients during the processing of *Cistanche deserticola*.

Thus, the objectives of the present study were to (1) provide useful information on the chemical changes in *Cistanche deserticola* during steaming and drying processes by using ultraperformance liquid chromatography-tandem mass spectrometry (UHPLC-MS/MS) combined with a widely targeted metabolomic approach; (2) identify the differential metabolites and their regulation rules, and reveal the possible conversion pathways in *Cistanche deserticola* during processing. This study is, therefore, expected to provide a theoretical reference for the formation mechanism of high-quality *Cistanche deserticola*.

## Materials and Methods

### Materials and Chemicals

Raw materials: Fresh *Cistanche deserticola* samples were obtained from the Hetian region in Xinjiang Province of China. The samples were carefully selected with the same size (average length, diameter, and weight were 11.7 ± 1.1 cm, 7.0 ± 1.1 cm, and 360 ± 8.9 g, respectively). The samples were stored at room temperature in a dark environment with an initial moisture content of about 78.56% ± 3.47%. Prior to the experiments, *Cistanche deserticola* samples were washed with tap water to remove the dust on the surface. Excess water on its surface was removed by blotting paper.

Chemicals: Methanol, acetonitrile, and formic acid were liquid-chromatography mass spectrometry grade (LC-MS) and purchased from Merck (Sigma Aldrich, MO, USA). The other analytical standards presented a purity higher than 98% (Sigma Aldrich, MO, USA).

### Experimental Design

Previous studies have shown that the chemical compounds distribute unevenly in the longitudinal direction of *Cistanche deserticola* (1). Therefore, to obtain the same initial contents of chemical compounds in each sample, in the present research all selected *Cistanche deserticola* was cut into three equal parts for the fresh group (A), steamed without drying group (B), and dried after steaming group (C), respectively, by longitudinal segmentation with the longitudinal symmetry axis as the center (20).

For group B, the samples were successively steamed for 8 min according to preliminary experiments. A pulsed vacuum steaming equipment (self-developed by China Agricultural University, Beijing, China) was used for steaming treatment of fresh *Cistanche deserticolas*. Steamed samples were dried in a vacuum freeze-dryer (LGJ-25C, Si Huan Scientific Instrument Factory Co., Beijing, China). The heating plate and cold trap temperature were 30 and −60°C, respectively. For group C, the samples were successively steamed using pulsed vacuum equipment for 8 min and dried in the hot air impingement dryer (self-developed by China Agricultural University, Beijing, China) until the final moisture content of 10% (w.b.). The airflow rate and temperature were set at 6 m/s and 60°C, respectively, referring to the research results of Zou et al. (11). All samples were stored at −20°C no more than 7 days before further analysis.

### Determination of Appearance Color of Cistanche Deserticola

The appearance color of the *Cistanche deserticola* before and after each thermal processing was measured using a colorimeter (SMY-2000SF, Shengming Yang Co., Beijing, China), and the blackness was characterized by L^*^ value.

### Sample Preparation and Extraction

The metabolite extraction was carried out according to the method reported previously by Chen et al. (21) with some minor modifications. In brief, the dried samples were crushed using a mixer mill (MM 400, Retsch Company, Haan, Germany) with a zirconia bead for 2 min at 60 Hz. Then 50 mg powder (sifted through a 65 mesh sieve) of each sample was precisely weighed, transferred to an Eppendorf tube, and extracted with 1 ml methanol/water mixture (v:v = 3:1). After 30 s vortex, the mixture was homogenized twice at 35 Hz for 4 min, sonicated for 15 min in an ice-water bath, and then shaken overnight at 4°C. After centrifugation at 12,000 rpm for 15 min at 4°C, the supernatant was collected and filtered through a 0.22-μm membrane, then the obtained extract was transferred to 2-ml glass vials and store at −80°C until the UHPLC-MS/MS analysis.

### Metabolites Analysis by UHPLC-MS

#### UHPLC Conditions

The UHPLC separation was carried out using an EXIONLC system (Sciex Technologies, Framingham, MA, USA). The analytical conditions were as follows: column: Waters ACQUITY UHPLC HSS T3 C18 (1.8 μm, 2.1 × 100 mm); solvent system: mobile phase A (0.1% formic acid in water) and mobile phase B (acetonitrile containing). The gradient program: 98% A/2% B at 0 min, 50% A/50% B at 10 min, 5% A/95% B at 11 min, 98% A/2% B at 13.1 min, and 98% A/2% B at 15 min. Flow rate: 0.40 ml/min; column temperature: 40°C; injection volume: 2 μl; automatic injection temperature: 4°C.

#### ESI-QTRAP-MS/MS Conditions

A triple quadrupole (QQQ)-linear ion trap mass spectrometer (QTRAP, API 6500 QTRAP UHPLC-MS/MS) + QQQ spectrometer equipped with an ESI turbo ion-spray interface (Sciex Technologies, Framingham, MA, USA) was applied for MS analysis. The analytical conditions were as follows: ion spray voltage: +5,500 V (positive ion mode)/−4,500 V (negative ion mode), curtain gas: 35 psi, source temperature: 400°C, ion source gas 1: 60 psi, ion source gas 2: 60 psi, declustering potential: ±100 V. QQQ scans were acquired as multiple reaction monitoring (MRM) experiments with collision gas (nitrogen) set to 5 psi.

### Qualitative and Quantitative Analysis of Metabolites

Qualitative and quantitative analyses of metabolites were performed according to the methods by Liu et al. (18). Primary and secondary mass spectrometry data were qualitatively analyzed based on the self-built human metabolome database (MWDB) (Metware Biotechnology Co., Ltd. Wuhan, China) and the public database. Meanwhile, to ensure the accuracy of the qualitative analysis of some substances, interferences from repeated signals of Na^+^, ${\text{NH}}_{4}^{+}$, K^+^ and ions, and repetitive signals of fragment ions derived from other relatively large molecules and isotope signals were removed during identification. Metabolite structural analysis was performed with reference to the public databases (Mass Bank, KNApSAcK, HMDB, MoTo DB, and METLIN).

Metabolite quantification was carried out using the MRM mode of the QQQ mass spectrometry. In the MRM mode, the precursor ions (parent ions) of the target substances and excluded ions corresponding to other substances with different molecular weights were screened first using the quadrupole rod to initially eliminate interference. The precursor ions then break through the collision chamber to form many fragment irons after ionization, which were filtered by QQQ to select single-fragment ions with the desired characteristics while eliminating interference from non-target ions. Finally, after obtaining the metabolite mass spectrometry data of different samples, the mass spectrum peaks of all substances were integrated, and the mass spectra peaks of the same metabolite in different samples were integrated and corrected using Multi Quant version 3.0.2 (ABSCIEX, Concord, Ontario, Canada). The corresponding relative metabolite contents were represented as chromatographic peak area integrals.

### Data Processing and Analysis

The metabolic data were processed using orthogonal partial least squares-discriminant analysis (OPLS-DA) and hierarchical cluster analysis (HCA). OPLS-DA was used to discriminate each group; it is more sensitive than other statistical methods to variables with low correlations (17). The OPLS-DA models were validated through a permutation analysis (200 times). The model was considered stable when the model parameters (R^2^ and Q^2^) were both close to 1. The variable importance projection (VIP) values of metabolites were calculated. Any metabolite with VIP values greater than 1.0 and *p*-values less than 0.05 were selected as biomarkers for each paired comparison between different thermal processing stages of *Cistanche deserticola*. The screening of different metabolites was visualized in the form of the volcano plot. Metabolites accumulation among different samples was analyzed by using the R package (www.rproject.org/). The Venn diagram was built according to the program web-based smart diagram® (https://cloud.smartdraw.com/). The commercial databases, such as Kyoto Encyclopedia of Genes and Genomes (KEGG) (https://www.kegg.jp/kegg/), Pub Chem (https://pubchem.ncbi.nlm.nih.gov/), the Small Molecule Pathway Database (SMPDB) (https://smpdb.ca/), and HMDB (https://hmdb.ca/), were used for enrichment analysis of differential metabolites and finding metabolic pathways.

## Results and Discussion

### Appearance Color Changes of Cistanche Deserticola During Thermal Processing

**Figure 1:**
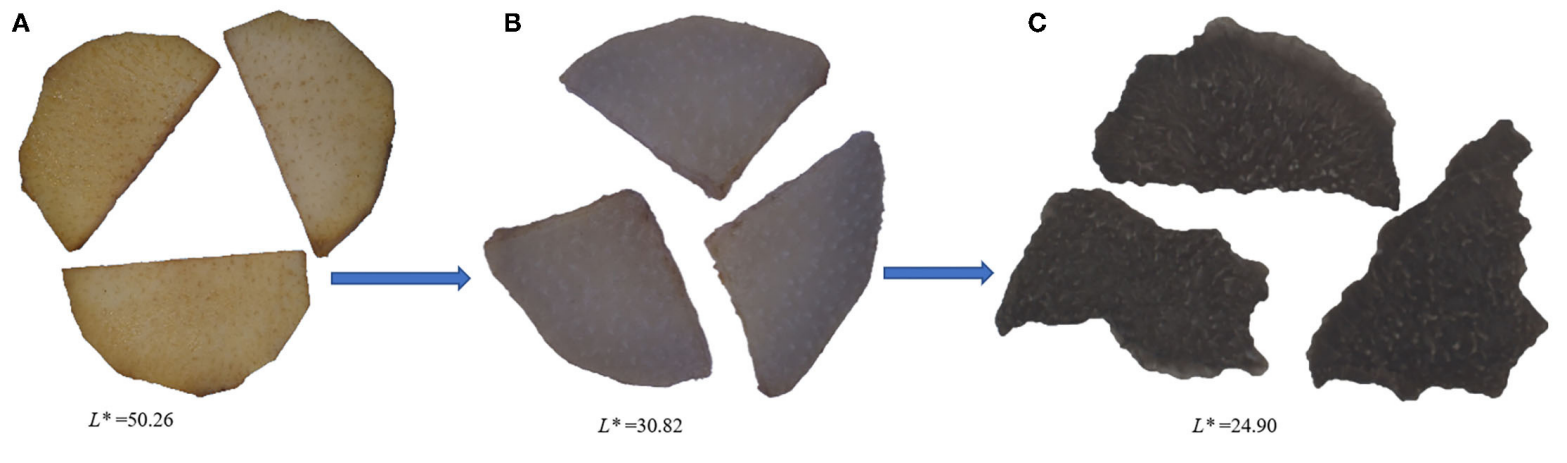
Appearance color characteristics during *Cistanche deserticola* thermal processing. **(A, B, C)** represent samples at the thermal processing stage before steaming, after steaming without drying, and after steaming and drying, respectively.

The difference in appearance color of *Cistanche deserticola* between the fresh, steamed and dried samples are representatively displayed in Figure 1. From fresh to dried sample with the going of the processing stage, the appearance color of the samples changed from yellow-brown to dark black, and the darkness in color became more and more obvious (the corresponding L^*^ value was reduced from 50.26 to 24.90). Obviously, the appearance changes of *Cistanche deserticola* mainly occurred in the steaming process. The Maillard reaction, during which sugars react with amino acids under thermal conditions (22), would be greatly responsible for the dark-colored appearance of processed rhizomes of *Cistanche deserticola*. Previous research studies have shown that precursors were converted into colorants and generated substances with dark color in the Maillard reaction (23). Similar findings were also observed in previous studies for steaming of *Polygonum multiflorum* (24) and rhizomes of *Polygonatum cyrtonema* (25). The darkness of the steamed samples was further deepened after drying. This phenomenon was probably due to the occurrence of a decrease in the pigment concentration during the drying process.

### Overview of the Metabolites in Raw and Thermal Processed Cistanche Deserticola Samples

**Figure 2:**
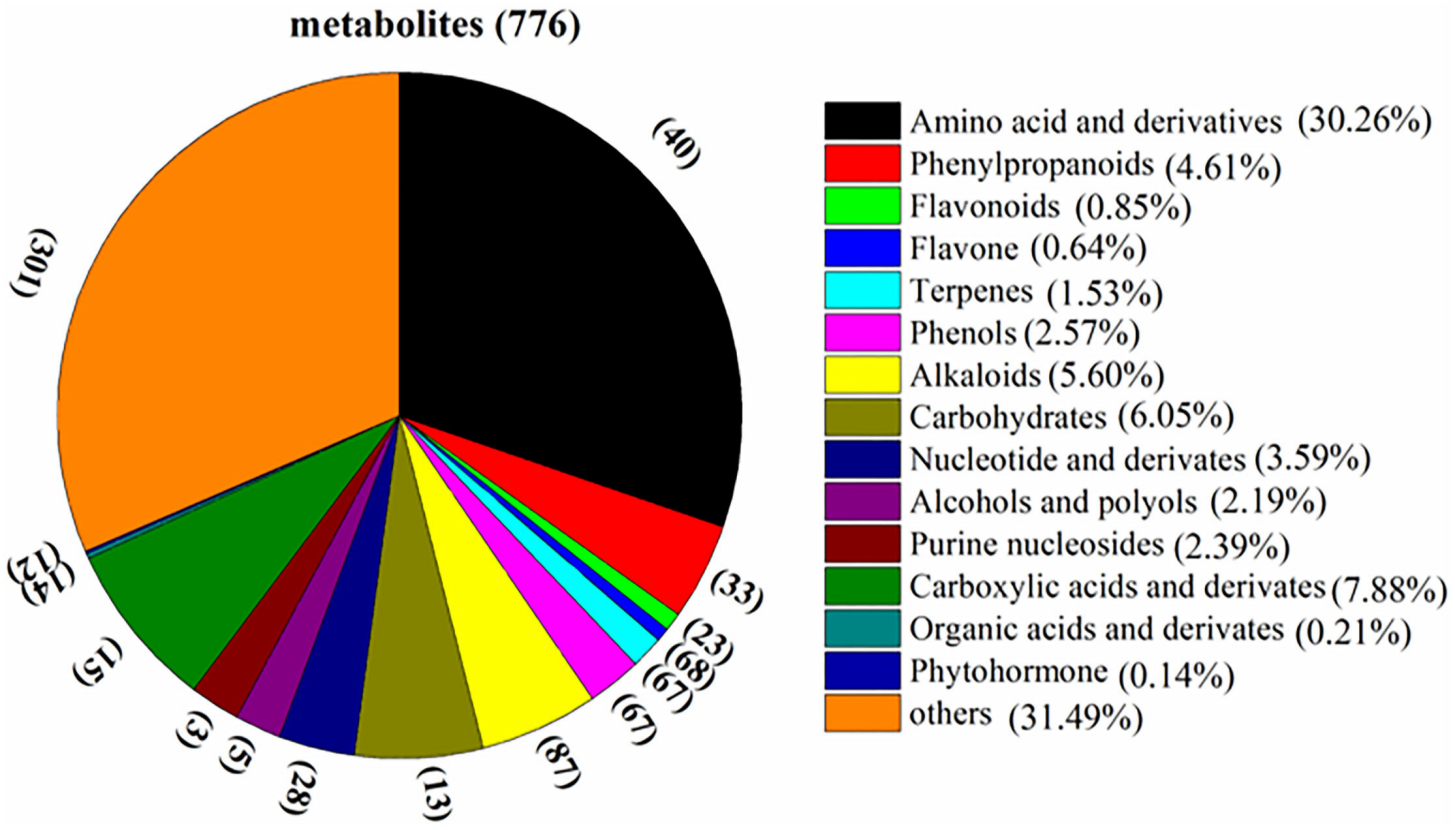
Classification and composition of the 776 metabolites of *Cistanche deserticola*.

The total ion chromatogram (TIC) of quality control (QC) sample (a mixture of all the samples investigated) and a multi-peak detection plot of chemicals in the MRM mode of the same sample are illustrated in Supplementary Figure 1. Different colored peaks represented different components in the sample. As shown in Figure 2, a total amount of 776 metabolites were identified in the current study (Supplementary Table 1) in the fresh *Cistanche deserticola* samples, which were divided into 15 classes, including 40 amino acid and derivatives, 33 phenylpropanoids, 23 flavonoids, 68 flavone, 67 terpenes, 67 phenols, 87 alkaloids, 13 carbohydrates, 28 nucleotide and derivatives, 5 alcohols and polyols, 3 purine nucleosides, 15 carboxylic acids and derivatives, 14 organic acids and derivatives, 12 phytohormone, and 28 other chemicals. Among them, the largest group was amino acid and derivatives, the relative content of which accounted for 30.26% of the total metabolite composition. In addition, 10 kinds of phenylethanoid glycosides, such as echinacoside and verbascoside, were detected and classified in the phenylpropanoids group.

**Figure 3:**
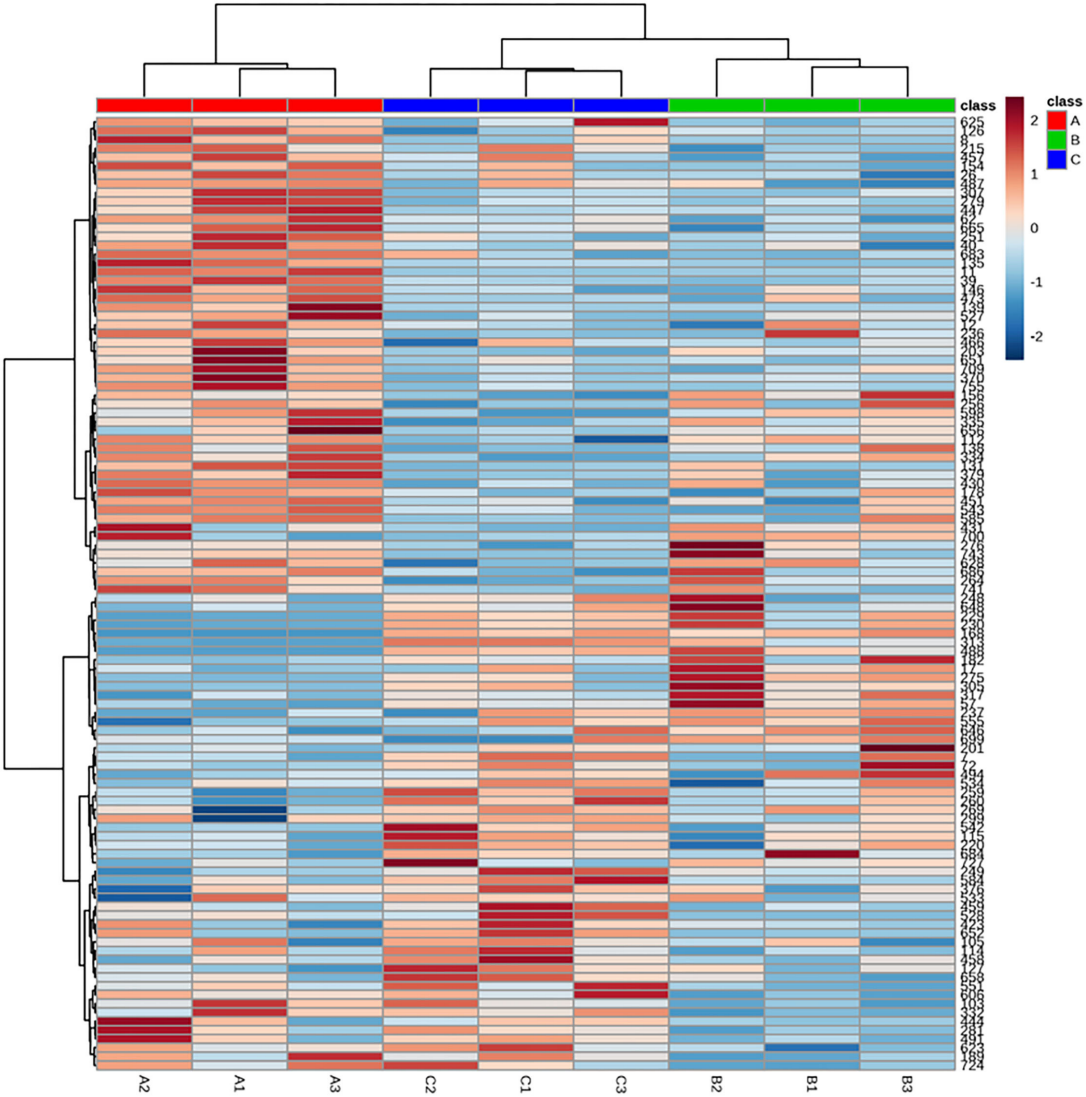
Heat map of the hierarchical clustering analysis of differential chemicals among samples of fresh (A), steamed (B), and dried after steaming (C).

The accumulation pattern of metabolites among different treatment groups was analyzed by HCA. As shown in Figure 3, 107 identified metabolites of *Cistanche deserticola* were clustered in heat maps based on Euclidean distance arithmetic. Metabolites identified at different thermal processing stages were gathered into three clusters according to the dendrogram. The brighter color indicates the higher content of a particular metabolite in the respective sample. The heat map of HCA showed larger di?erences in abundance between the fresh and steamed samples than those between steamed and dried samples, indicating that metabolites in *Cistanche deserticola* may have different transformations during the steaming and drying stage, and the types and quantities of metabolites involved in the steaming process are more than those in the drying process.

### Differential Metabolite Analysis of Cistanche Deserticola at Different Thermal Processing Stages

**Figure 4:**
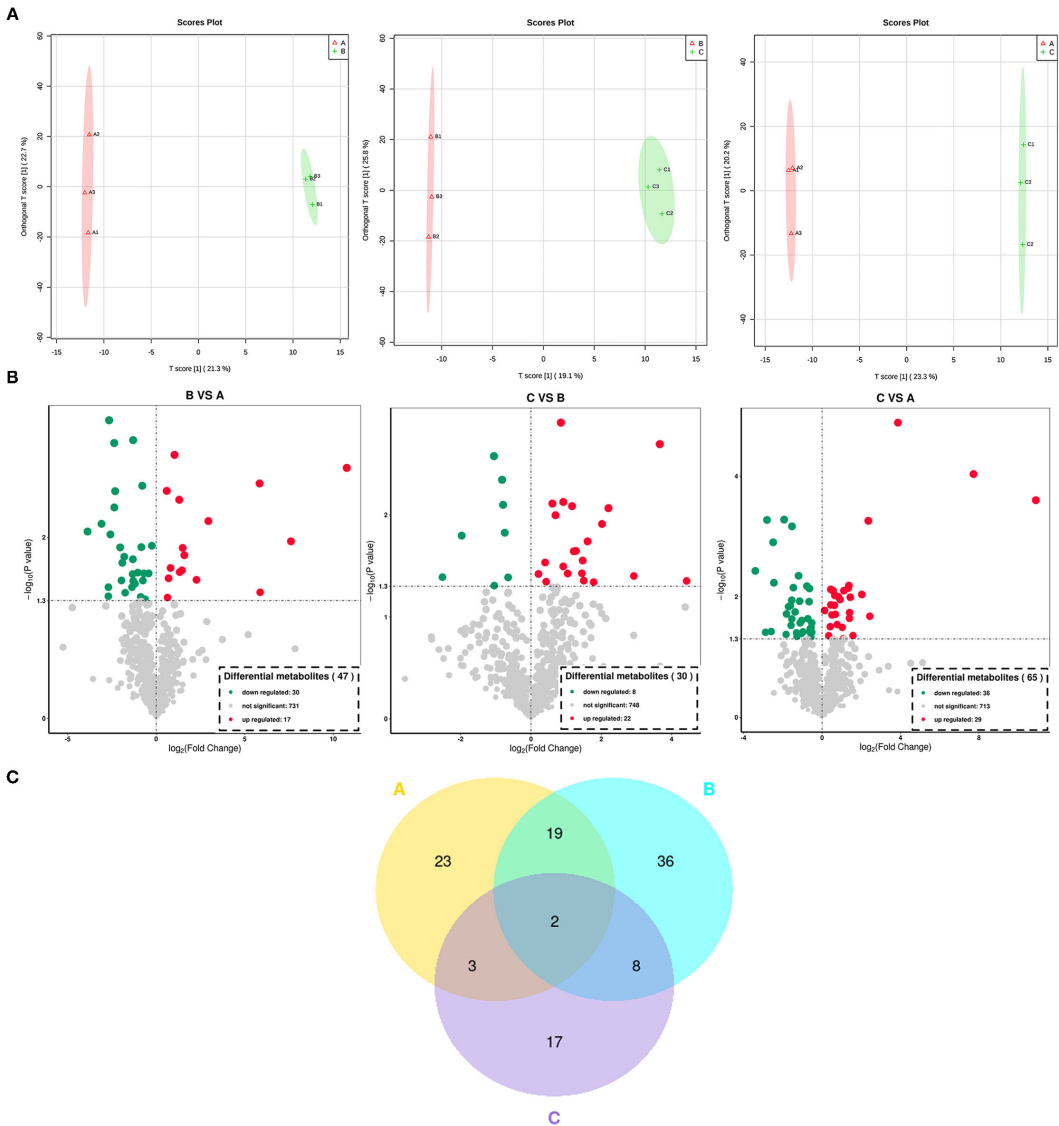
Differential metabolite analysis in all *Cistanche deserticola* samples processed at different stages. **(A)** the score plots of the differential metabolites generated from OPLS-DA; **(B)** the volcano plot of the differential metabolite in different samples; **(C)** the Veen diagram of the differential metabolite in different samples. A, fresh; B, steamed without drying; C, dried after steaming. OPLS-DA, orthogonal partial least squares discriminant analysis.

For a better understanding of the impact of each processing on the metabolites of *Cistanche deserticolas*, the OPLS-DA scatter scores of pairwise comparison groups are shown in Figure 4A, showing that the fresh, steamed, and dried after steaming *Cistanche deserticolas* were significantly different. Moreover, the R^2^Y and Q^2^ (as shown in Supplementary Figure 2) with high-test values indicated that this model was highly reliable without overfitting.

To screen the expression level of metabolites between the fresh, steamed, and dried after steaming *Cistanche deserticola*, the analysis of volcano plot was further applied among all 776 metabolites identified according to the fold-change, combined with VIP values to screen the differentially expressed metabolites. Significant differential metabolites were selected according to the criterion that a fold change score of ≥2 or ≤ 0.5 with a VIP ≥ 1. The screening results are illustrated in Figure 4B. In the volcanic map, each point represents a metabolite and the color of the scattered dots represents the final screening result. Red represents metabolites that are significantly upregulated (UR), green represents those significantly downregulated (DR), and gray represents those insignificantly different. As shown in Figure 4B, 47 metabolites in the fresh vs. steamed group (17 UR and 30 DR), 30 metabolites in steamed vs. dried group (22 UR and 8 DR), and 65 metabolites in the fresh vs. dried group (29 UR and 36 DR) were selected to be significantly differential. The number of significantly different metabolites in the fresh vs. steamed group was higher than those in the steamed vs. dried group, indicating that the influence on metabolites in the steaming process is higher than that of the drying process. The differential metabolites produced during thermal processing of *Cistanche deserticola* were further classified and compared. These differentially expressed metabolites were classified into 21 classes, mainly amino acids and their derivatives, flavonoids and their derivatives, phenylpropanoids, alkaloids, terpenes, phenols, and nucleotide and their derivatives (Table 1). In fresh vs. steamed group, it can be found that flavonoids (such as isoquercitrin, troxerutin, cyanidin, and fisetin), phenylpropanoids (such as chlorogenic acid and 3-(3,4-Dihydroxy-5-methoxy)-2-propenoic acid), and nucleotide and their derivatives (uracil and beta-Nicotinamide mononucleotide) were significantly DR, while amino acids and their derivatives (such as N6-Acetyl-L-lysine, 1-Methy-L-histidine, and L-Phenylalanine) were significantly UR. However, in the steamed vs. dried group, the expression trends of these types of differential metabolites were opposite. Some amino and their derivatives (such as N, N-Dimethylglycine), nucleotide and their derivatives (such as 2′-Deoxyuridine; Deoxyuridine) were significantly DR, while most of the phenols (such as methyl gallate and 4′-Prenyloxyresveratrol), flavonoids (such as isoquercitrin and cyanidin), phenylpropanoids (verbascoside), and terpenes (such as terpinolene and furanodiene) were significantly UR.

**Table 1:** List of significantly different metabolites up/downregulated in *Cistanche deserticola* under different thermal processing stages.

**KEGG-ID**	**Molecular mass**	**Metabolite name**	**Class**	**VIP**	***P*-value**	**Fold change**	**Regulation**
Thermal processing stage: steaming							
	101.05	1-Aminocyclopropanecarboxylic acid	Phytohormone	2.00	0.01	0.07	Downregulated
C05623	464.09	Isoquercitrin	Flavonoids	2.01	0.01	0.12	Downregulated
C06802	645.25	Acarbose	Alkaloids	1.79	0.04	0.15	Downregulated
C10526	286.12	(–)-Sativan	Flavonoids	1.84	0.04	0.15	Downregulated
	742.23	Troxeruti	Flavonoids	2.13	0.00	0.16	Downregulated
C16959	216.15	Furanodiene	Sesquiterpenoids	1.98	0.01	0.17	Downregulated
C08493	145.05	Indole-3-carboxaldehyde	Phytohormone	2.12	0.00	0.19	Downregulated
C12634	610.15	Kaempferol3-O-beta-sophoroside	Flavonoids	2.04	0.00	0.19	Downregulated
C05905	286.05	Cyanidin	Flavonoids	2.06	0.00	0.20	Downregulated
C09372	367.11	(+)-Bicuculline	Alkaloids	1.95	0.01	0.24	Downregulated
C00106	112.03	Uracil	Nucleotide and derivatives	1.86	0.03	0.26	Downregulated
C00852	354.10	Chlorogenic acid	Phenylpropanoids	1.93	0.02	0.27	Downregulated
C00455	334.06	beta-Nicotinamide mononucleotide	Nucleotide and derivatives	1.94	0.02	0.29	Downregulated
C01965	290.14	Trimethoprim	Phenol ethers	1.82	0.04	0.29	Downregulated
C10414	268.07	Dalbergin	Coumarins	1.82	0.03	0.39	Downregulated
C12312	133.05	Indolin-2-one	Alkaloids	1.89	0.02	0.39	Downregulated
C00345	276.02	6-Phosphogluconic acid	Organooxygen compounds	1.93	0.02	0.40	Downregulated
C12298	198.16	Citronellyl acetate	Monoterpenoids	2.12	0.00	0.41	Downregulated
C01118	219.07	O-Succinyl-L-homoserine		1.86	0.03	0.41	Downregulated
C10851	175.08	Calystegine B2	Alkaloids	1.86	0.03	0.44	Downregulated
C01378	288.06	Fisetin	Flavonoids	1.88	0.02	0.49	Downregulated
C06575	134.11	P-Cymene	Monoterpenoids	1.77	0.05	0.54	Downregulated
C05123	125.99	2-Hydroxyethanesulfonate	Organic acids	1.96	0.01	0.56	Downregulated
C18326	234.14	N-p-Coumaroyl putrescine	Phenolamides	1.77	0.04	0.56	Downregulated
C05610	208.07	Trans-3,5-Dimethoxy-4-hydroxy cinnamaldehydee	Phenylpropanoids	2.07	0.00	0.58	Downregulated
C05619	210.05	3-(3,4-Dihydroxy-5-methoxy)-2 propenoic acid	Cinnamic acids and derivatives	1.83	0.03	0.59	Downregulated
C07650	263.07	Gemcitabine	Pyrimidine nucleosides	1.88	0.03	0.62	Downregulated
C02107	150.02	D-tartaric acid	Organic acids and derivatives	1.76	0.05	0.65	Downregulated
C09922	386.10	Cleomiscosin A	Coumarins	1.88	0.02	0.74	Downregulated
C00568	137.05	P-Aminobenzoate	Benzoic acid derivatives	1.95	0.01	0.84	Downregulated
C02727	188.12	N6-Acetyl-L-lysine	Amino acid and derivatives	2.07	0.00	1.51	Upregulated
C17756	151.06	Leukoaminochrome	Indoles and derivatives	1.76	0.04	1.56	Upregulated
C11045	294.12	Aspartame	Carboxylic acids and derivatives	1.85	0.03	1.62	Upregulated
C05138	332.24	17a-Hydroxypregnenolone	Steroidsand steroid derivatives	1.89	0.02	1.76	Upregulated
C00255	376.14	Riboflavine	Vitamins	2.11	0.00	2.05	Upregulated
C10372	272.10	9-Methoxy-alpha-lapachone	Quinones	2.05	0.00	2.47	Upregulated
C10875	412.12	Podophyllotoxinone	Lignans	1.91	0.02	2.51	Upregulated
C01152	169.09	1-Methy-L-histidine	Amino acids	1.88	0.02	2.75	Upregulated
C09274	310.20	Tabernanthine	Alkaloids	1.95	0.01	2.82	Upregulated
C00079	165.08	L-Phenylalanine	Amino acid and derivatives	1.93	0.02	3.00	Upregulated
C05198	251.10	5'-Deoxyadenosine	Nucleotide and derivatives	1.84	0.03	4.84	Upregulated
C08431	251.10	Cordycepin	Nucleotide and derivatives	1.84	0.03	4.84	Upregulated
C00153	122.05	Nicotinamide	Alkaloids	2.01	0.01	7.72	Upregulated
C02353	329.05	Adenosine 2',3'-cyclic phosphate	Purine nucleotides	2.07	0.00	57.29	Upregulated
C00942	345.05	Guanosine 3',5'-cyclic monophosphate	Nucleotide and derivatives	1.78	0.04	58.52	Upregulated
C10190	372.12	Tangeretin	Flavonoids	1.97	0.01	195.63	Upregulated
	374.28	Ginkgolic acid C17:1	Phenols	2.10	0.00	1737.4	Upregulated
Thermal processing stage: drying							
C05243	299.15	N-Methylcoclaurine	Alkaloids	1.87	0.04	0.17	Downregulated
C01026	103.06	N,N-Dimethylglycine	Amino acid and derivatives	2.03	0.01	0.25	Downregulated
C09202	376.15	Tripdiolide	Diterpenoids	2.20	0.00	0.48	Downregulated
C09868	150.10	(R)-Menthofuran	Prenol lipids	1.89	0.04	0.48	Downregulated
C05380	180.05	Nicotinurate	Carboxylic acids and derivatives	2.15	0.00	0.56	Downregulated
C17496	350.25	10-Gingerol	Phenols	2.09	0.01	0.57	Downregulated
C02666	178.06	Coniferylaldehyde	Phenylpropanoids	2.03	0.01	0.59	Downregulated
C00526	228.07	2'-Deoxyuridine	Nucleotide and derivatives	1.94	0.04	0.64	Downregulated
C10501	624.21	Verbascoside	Phenylpropanoids	1.92	0.04	1.16	Upregulated
C00576	101.08	Betaine aldehyde	Organonitrogen compounds	1.99	0.03	1.32	Upregulated
	184.04	Methyl gallate	Phenols	1.88	0.04	1.35	Upregulated
C08316	338.32	Erucic acid	Fatty Acyls	2.11	0.001	1.53	Upregulated
C10283	312.14	4'-Prenyloxyresveratrol	Phenols	2.07	0.01	1.62	Upregulated
C05905	286.05	Cyanidin	Flavonoids	2.21	0.00	1.81	Upregulated
C17497	194.09	Zingerone	Phenols	1.96	0.03	1.89	Upregulated
C00153	122.05	Nicotinamide	Alkaloids	2.13	0.01	1.89	Upregulated
C00881	227.09	Deoxycytidine	Nucleotide and derivatives	1.95	0.04	2.06	Upregulated
	174.10	6(1H)-Azulenone, 2,3-dihydro-1,4-dimethyl	Miscellaneous	2.09	0.01	2.25	Upregulated
C06075	136.13	Terpinolene	Monoterpenoids	2.01	0.02	2.32	Upregulated
	804.38	Rebaudioside B	Diterpenoids	2.03	0.02	2.43	Upregulated
C07650	263.07	Gemcitabine	Pyrimidine nucleosides	1.90	0.04	2.73	Upregulated
C00328	208.08	L-Kynurenine	Amino acid and derivatives	2.00	0.03	2.77	Upregulated
C10333	367.16	Isatidine	Alkaloids	1.92	0.04	2.83	Upregulated
C09770	334.07	Cedeodarin	Flavonoids	2.06	0.02	3.06	Upregulated
C13202	472.39	DL-alpha-Tocopherylacetate	Phenols	1.86	0.04	3.44	Upregulated
C04294	143.04	4-Methyl-5-thiazoleethanol	Azoles	2.04	0.01	4.06	Upregulated
C10640	372.16	Kadsurin A	Lignans	2.13	0.01	4.61	Upregulated
C16959	216.15	Furanodiene	Sesquiterpenoids	1.87	0.04	7.61	Upregulated
C16968	366.11	Neoglycyrol	Coumarins	2.21	0.00	12.69	Upregulated
C05623	464.10	Isoquercitrin	Flavonoids	1.85	0.04	21.52	Upregulated

*VIP, variable importance projection*.

These results showed that the chemical composition of *Cistanche deserticola* has undergone conversion during thermal processing, which is mainly reflected in the conversion of flavonoids, phenylpropanoids, and amino acids, and the conversion mechanism of these components is different in different processing stages. The use of a high temperature during the steaming and drying processes was previously found to promote hydrolysis, redox, isomerization, substitution, and other thermophysical and chemical reactions of metabolites (26). In this study, it was found that the metabolites, such as flavonoids and phenylpropanoids, were significantly accumulated in the steamed *Cistanche deserticola* compared to their corresponding fresh one, indicating that some key physiological and metabolic activities leading to the synthesis of flavonoids and phenylpropanoids might be activated under high temperature and humidity. This result can also be supported by the report from Peng et al. (10) who found that the content of PhGs (belonging to phenylpropanoids) increased after steaming. However, the accumulation of these components in the dried sample after steaming showed a significant decrease, which may be attributed to the thermal degradation of these heat-sensitive components during the long-term drying process. Previous studies have shown that flavonoid glycosides can be decomposed into sugar bodies and flavonoid aglycones under thermal conditions, and flavonoids loss during the drying process was synthetically affected by temperature and drying time (26, 27). The upregulation of amino acids and their derivatives (N6-Acetyl-L-lysine, 1-Methy-L-histidine, and L-Phenylalanine) is attributed to the high-temperature-promoting protein degradation during steaming processing. In addition, it was also observed that some other amino acids and their derivatives of N, N-Dimethylglycine, L- Kynurenine, glycine, serine, and threonine were DR. The decrease in the content of these amino acids might be associated with thermal-induced Maillard reaction during which reducing sugars react with amino acids to generate 5-HMF, contributing to the production of black appearance in *Cistanche deserticola* (22). The results of Section appearance color changes of cistanche deserticola during thermal processing further verified this hypothesis. Therefore, the blackening of *Cistanche deserticola* during steaming was probably related to the metabolism of amino acids.

Venn diagram was used to differentiate the common and exclusive metabolites of *Cistanche deserticola* during different thermal processing stages. As shown in Figure 4C, both common and unique metabolites exist between the different comparison groups. Twenty-one common metabolites were observed between the fresh and steamed group, while only 5 and 10 metabolites were found common between the fresh and dried group, steamed and dried group, respectively. Thus, a total of 23 and 17 exclusive metabolites (*p* < 0.05) were observed in *Cistanche deserticola* during the thermal processing stage of steaming and drying, respectively. This result further confirmed that steaming was particularly critical for the conversion of metabolites during *Cistanche deserticola* processing.

### Enrichment Analysis and KEGG Pathway Impact Analysis of Differential Metabolites

**Figure 5:**
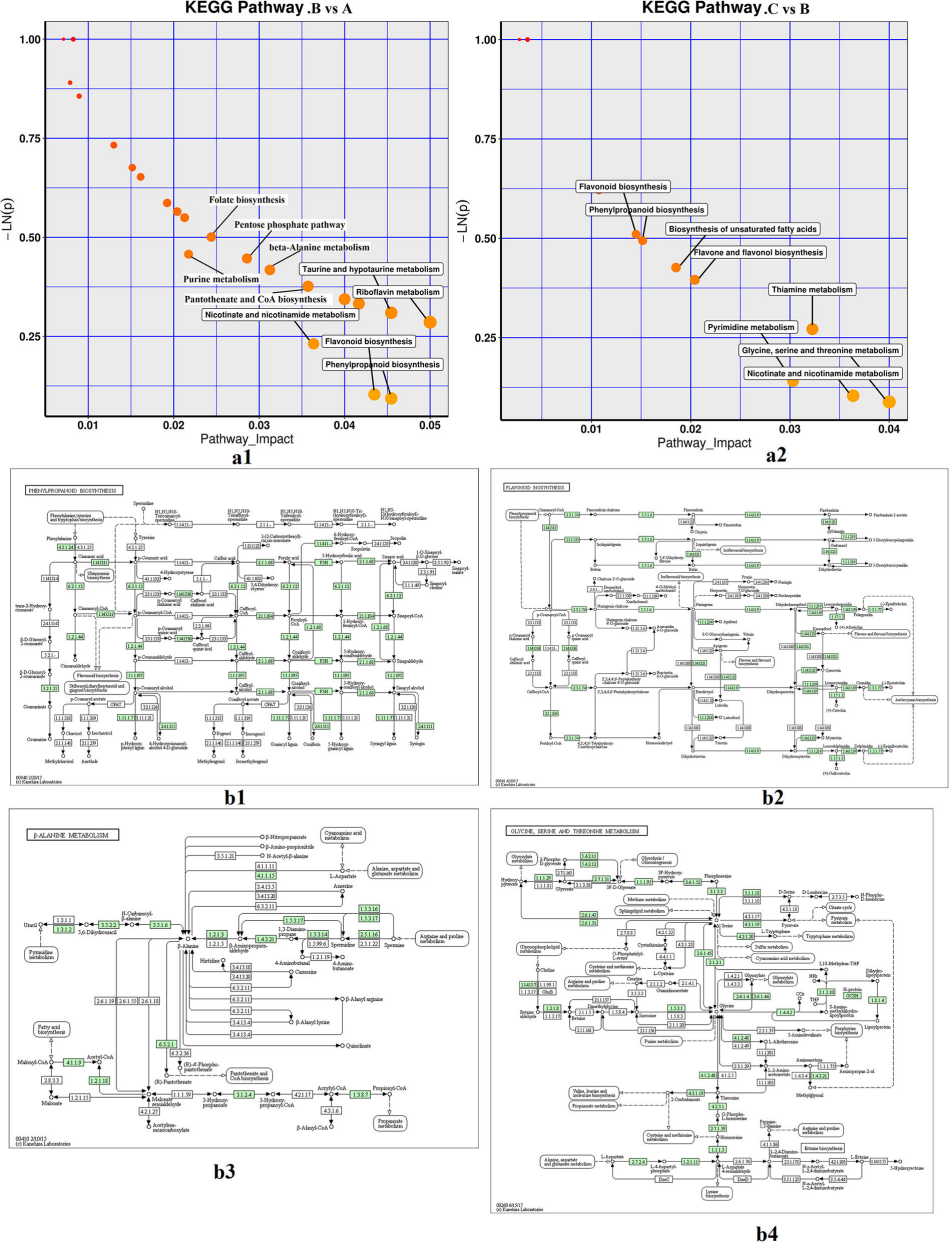
Metabolic enrichment pathway analysis in two comparative groups **(a1,a2)** and important KEGG pathway maps **(b1,b4)**. **(a1,a2)** represent the enrichment analysis of different metabolites in the steaming and drying processes, respectively; **(b1,b4)** respectively represent phenylpropanoid biosynthesis pathway, flavonoid biosynthesis pathway, alanine metabolism pathway, glycine, serine and threonine metabolism pathway. A, fresh; B, steamed without drying; C, dried after steaming. KEGG, Kyoto Encyclopedia of Genes and Genomes.

The differential metabolites (*p* < 0.05) in fresh and processed samples were mapped to the KEGG, HMDB, and PubChem online databases, which contain knowledge of the molecular interaction, reaction, and relation networks, and the enrichment results and detailed metabolic pathways are shown in Supplementary Table 2 and Figure 5. As shown in Figures 5a1,a2, pathway impact revealed the enrichment of phenylpropanoid biosynthesis, flavonoid biosynthesis, alanine metabolism, riboflavin metabolism, taurine and hypotaurine metabolism, and nicotinate and nicotinamide metabolism during steaming of *Cistanche deserticola*. Whereas, during the drying process after steaming, the metabolic pathways of the differential metabolites mainly contained glycine, serine and threonine metabolism, thiamine metabolism, pyrimidine metabolism, and unsaturated fatty acids biosynthesis. Furthermore, some metabolic pathways between these two pairwise comparisons overlapped, such as nicotinate and nicotinamide metabolism, phenylpropanoid biosynthesis, and flavonoid biosynthesis, but their enrichment levels were very different in two pairwise comparisons. These results suggested that the conversion pathways of metabolites between the steaming and drying processes of *Cistanche deserticola* were different, and the differences in metabolic pathways could explain the differences in the presence of differentially exclusive metabolites during thermal processing. These biochemical alterations might be used to comprehend the impact of thermal processing stages on *Cistanche deserticola* composition.

Based on the KEGG annotation and enrichment analysis, four metabolic pathways (phenylpropanoid biosynthesis, flavonoid biosynthesis, alanine metabolism, and glycine, serine, and threonine metabolism) were chosen as key metabolites to characterize the conversion of the main active components of *Cistanche deserticola* during thermal processing (Figures 5b1,b2). The current study indicated that phenylpropanoids and flavonoids were accumulated but amino acids were degraded in steamed *Cistanche deserticola* compared to fresh and dried samples. The phenylpropanoid biosynthetic pathway is upstream of biosynthetic pathway of flavonoid. Similar conclusions were published by Liu et al. (18) who reported that the accumulation level of phenylpropanoids in the process of rice yellowing has increased significantly, compared with normal rice. Phenylpropanoids are derived from cinnamic acid, and their precursor is phenylalanine, which can be synthesized by activating the activity of phenylalanine ammonia-lyase (PAL) when heated (28). Previous studies reported that the phenylpropanoid pathway led to the biosynthesis of coumarins, flavones, isoflavones, and flavanols, which are the important weapons for plant defense (29), and to prevent cell death caused by the strong heat stress in the steaming process, the phenylpropanoid pathway may be enhanced due to the biological stress caused by high temperature (30, 31). Flavonoids are the main secondary metabolites derived from phenylpropanoids (32), and their accumulation could protect plants from oxidative damage by scavenging-free radicals (33). Compared to the fresh and dried *Cistanche deserticola*, the higher biosynthesis of flavonoids in the steamed *Cistanche deserticola* may be associated with enhanced heat stress during the steaming process providing protection against reactive oxygen species (ROS) (34, 35). As shown in Figures 5b3,b4, amino acid metabolism played an important role in the thermal processing of *Cistanche deserticola*. Content changes of alanine, glycine, serine, and threonine after steaming found in medicinal herbs have been used to indicate the occurrence of the Maillard reaction (36). Nevertheless, due to the complicated process of the *Cistanche deserticola* steaming, a comprehensive evaluation of the *Cistanche deserticola* steaming, such as blackening in appearance, active compounds, and metabolic biomarkers, should be further investigated.

## Conclusions

In the present study, UHPLC-MS/MS-based widely targeted metabolomics approach was employed to study the formation mechanism of active compounds at different thermal processing stages of *Cistanche deserticola*. The current results revealed that the biosynthesis of some key metabolites, such as phenylpropanoids and flavonoids, was significantly enhanced during the steaming process. The expression level of amino acids in steamed *Cistanche deserticola* was enhanced, indicating the transformation between primary and secondary metabolites. In addition, the blackening of the appearance of *Cistanche deserticola* mainly occurred in the steaming stage rather than the drying stage, this characteristic is associated with the amino acids' metabolism pathway. However, the levels of the above metabolites decreased significantly during the drying process, suggesting the formation of active compounds mainly occurred in the steaming stage during the thermal processing of *Cistanche deserticola*. To the best of our knowledge, this is the first time that the widely targeted metabolomic method was used to reveal that the mechanism of active compounds changes during the thermal processing and their crucial contribution to the *Cistanche deserticola* blackening. However, further investigation is needed for a better understanding of the relationship between the biosynthesis of active compounds and the blackening of the appearance during thermal processing.

## Data Availability Statement

The original contributions presented in the study are included in the article/Supplementary Material, further inquiries can be directed to the corresponding author/s.

## Author Contributions

ZA conducted experimental design, performed the experiments, generated the data, and wrote this manuscript. YZ performed the metabolomics analysis. XL provided the statistical analysis. WS conducted data processing and investigation. Funding acquisition, overall framework, and writing-reviewing were completed by YL. All authors contributed to the article and approved the submitted version.

## Funding

This work was financially supported by the Department of Science and Technology of Guangdong Province (No. 2018B020241003).

## Conflict of Interest

The authors declare that the research was conducted in the absence of any commercial or financial relationships that could be construed as a potential conflict of interest.

## Publisher's Note

All claims expressed in this article are solely those of the authors and do not necessarily represent those of their affiliated organizations, or those of the publisher, the editors and the reviewers. Any product that may be evaluated in this article, or claim that may be made by its manufacturer, is not guaranteed or endorsed by the publisher.
