# Supplementary material for: Widely Targeted Metabolomics Analysis to Reveal Transformation Mechanism of Cistanche Deserticola Active Compounds During Steaming and Drying Processes
Source: Front Nutr. 2021 Oct 14;8:742511. doi: 10.3389/fnut.2021.742511 (PMC8551385; doi:10.3389/fnut.2021.742511)
Supplement: Supplementary file 1 [file Data_Sheet_1.docx]

Supplementary Fig. S1. Total iron current chromatogram of a quality control (QC) samples by MS under the multiple reaction monitoring (MRM) mode (B and C for positive mode and negative mode, respectively).


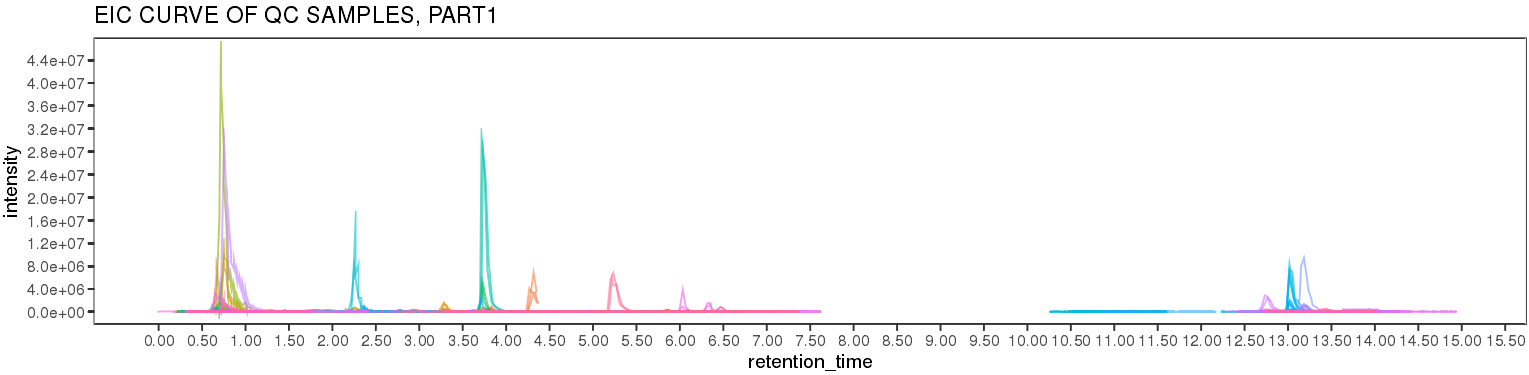

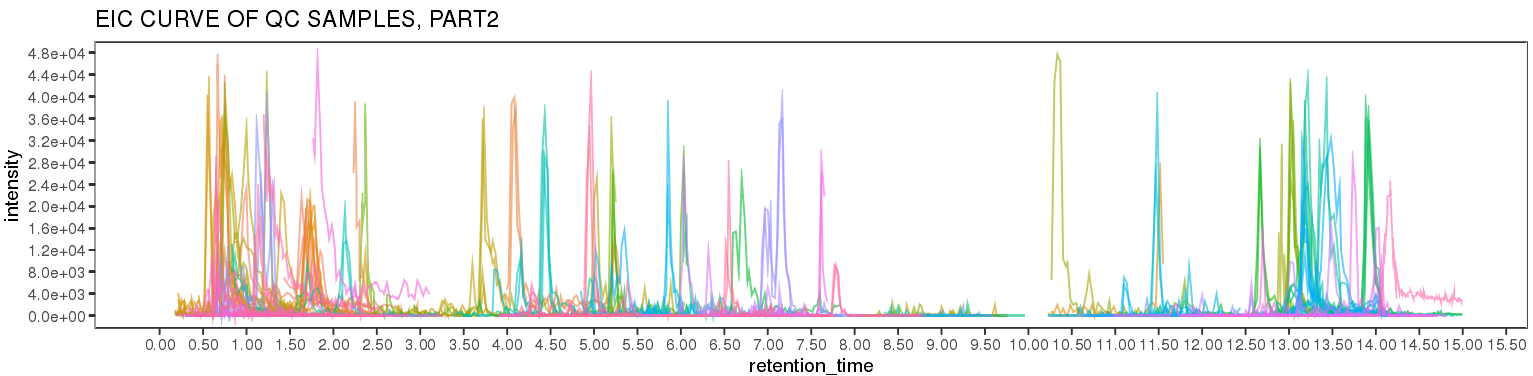

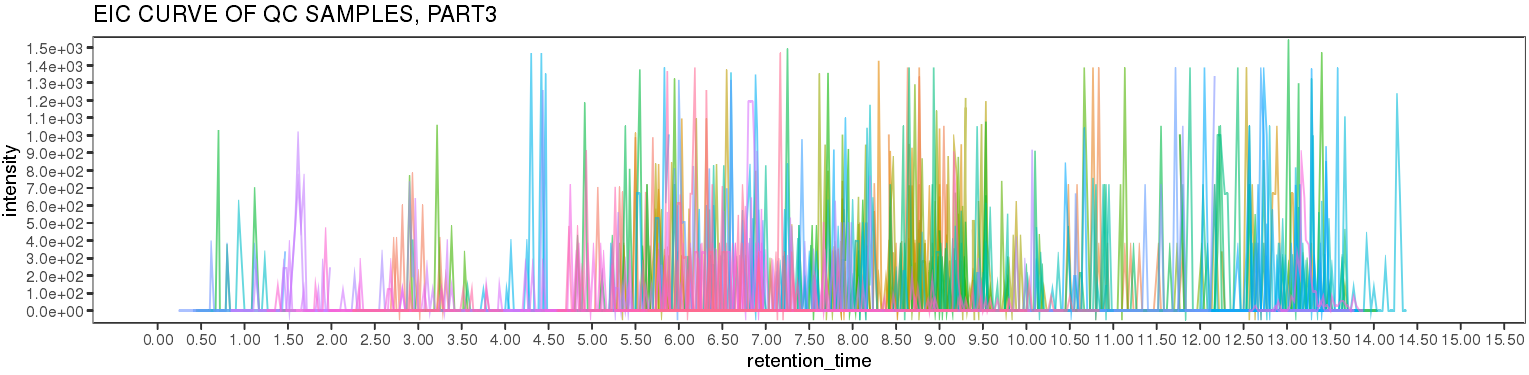


**A**

**B**

**C**

Supplementary Fig. S2. Permutation test for OPLS-DA model for pairwise comparison groups during thermal processing (A: fresh vs steamed group; B: steamed vs dried after steaming group; C: fresh vs dried after steaming group).


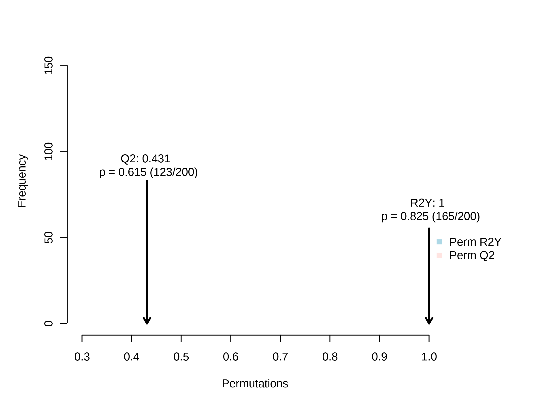

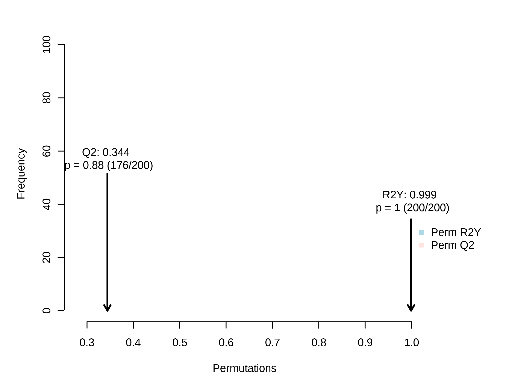

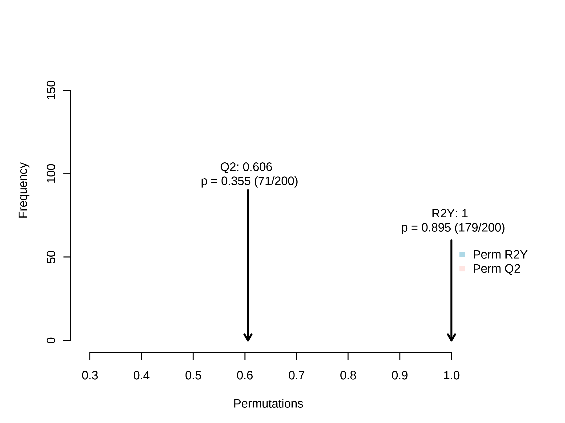


**A**

**B**

**C**
